# The application and challenges of ChatGPT in laboratory medicine

**DOI:** 10.1515/almed-2025-0080

**Published:** 2025-08-15

**Authors:** Zhili Niu, Xiandong Kuang, Juanjuan Chen, Xin Cai, Pingan Zhang

**Affiliations:** Department of Clinical Laboratory, Institute of Translational Medicine, 117921 Renmin Hospital of Wuhan University, Wuhan, Hubei, P.R. China

**Keywords:** ChatGPT, chatbots, medical laboratory, advantages, challenges

## Abstract

In recent years, with the rapid development of artificial intelligence technology, chatbots have demonstrated significant potential in the medical field, particularly in medical laboratories. This study systematically analyzes the advantages and challenges of chatbots in this field and delves into their potential applications in disease diagnosis. However, the reliability and scientific nature of chatbots are influenced by various factors, including data quality, model bias, privacy protection, and user feedback requirements. To ensure the accuracy and reliability of output content, it is essential to not only rely on legal frameworks such as the EU AI Act for necessary protection but also to employ two assessment tools, METRICS and CLEAR. These tools are designed to comprehensively evaluate the quality of AI-generated health information, thereby providing a solid theoretical foundation and support for clinical practice.

## Introduction

Artificial intelligence (AI), first conceptualized in 1950 by Alan Turing’s seminal question, “Can machines think?”, laid the theoretical groundwork for this field [1]. The term “artificial intelligence” was officially coined in 1956 by John McCarthy and colleagues at the Dartmouth Conference, marking the birth of AI as an independent research discipline [1]. AI spans a broad spectrum, from general artificial intelligence (AGI), which seeks to replicate human intelligence, to narrow artificial intelligence (ANI), focused on specific tasks. Initially, AI relied on rule-based algorithms, such as “if-then” rules, with these non-adaptive systems still in use in many clinical settings today. However, technological advancements have shifted AI applications toward more sophisticated methods, including machine learning (ML) and deep learning (DL) algorithms, significantly enhancing data analysis, image recognition, and disease prediction in medical diagnostics.

With clinical laboratories gradually achieving automation, the massive and complex test results generated daily exceed the processing capacity of a single human brain, making the application of AI increasingly important. AI can deeply analyze high-quality structured data in clinical laboratories, driving the transformation of diagnostic methods [2]. Generative AI foundational models can simplify workflows, improve efficiency, and reduce healthcare costs; enhance personalized medicine by improving the precision of disease risk and outcome predictions; and improve public health literacy by empowering patients with easily understandable health information for better self-management [3].

In recent years, the development of large language models (LLMs), such as pre-trained transformers (GPT), BERT, and the Pathways language model (PaLM), has had a profound impact on various fields, including text generation, machine translation, and creative content design. LLMs have revolutionized patient-physician interactions by simulating human-like conversations, significantly altering traditional communication patterns in healthcare [4]. Although AI chatbots have the potential to enhance medical education, promptly address routine questions about laboratory tests, and assist in interpreting test results, their applications remain in their early stages. The associated risks and challenges demand further exploration [5], 6]. Patients often seek information on the internet when timely explanations of laboratory test results are not provided, potentially leading to misinformation and health risks [7], [8], [9]. Furthermore, the performance of different chatbot models varies significantly, influenced by factors such as model configurations, prompt diversity, and evaluation methodologies [10], [11], [12]. AI chatbots may generate seemingly credible but actually inaccurate information, necessitating a comprehensive understanding of their strengths and limitations by clinicians and laboratory personnel to effectively utilize and manage them [13], 14].

The purpose of this review is to summarize the advantages and limitations of chatbots in medical laboratories and to explore the challenges and future research directions in clinical laboratory settings. It aims to provide valuable references for clinical laboratory staff applying chatbots, thereby promoting the further application of artificial intelligence technology in the medical field.

## Demands and current status of artificial intelligence

Laboratory medicine reports play a crucial role in clinical decision-making, significantly influencing diagnosis and treatment choices while also playing a vital role in patient health management. However, the complexity of these reports often confuses non-experts, leading many patients to seek explanations from chatbots when facing health issues. Research indicates that 78 % of ChatGPT users tend to utilize it for self-diagnosis, underscoring the significant demand for reliable sources of health information [15]. According to studies by Giardina et al., 46 % of patients resort to web searches to understand their test results, yet this behavior also reflects potential challenges they face in assessing the severity of these results [5]. The absence of timely and clear diagnostic information can exacerbate patient anxiety and misunderstanding. Furthermore, research by Kopanitsa demonstrates that patients receiving automatically generated explanations are significantly more likely to follow up (71 %) compared to those who only receive test results (49 %). Patients generally appreciate the timeliness of these explanations, highlighting how innovative communication methods can greatly enhance patient experiences and health outcomes [16].

Significant hierarchical characteristics are observed in the perception and acceptance of AI among clinical staff [17], [18], [19]. A majority of respondents support AI as a supplementary tool, particularly valuing its role in data analysis. Young practitioners (<45 years old) exhibit relatively lower self-assessed AI knowledge levels compared to their peers, yet they demonstrate a significantly higher inclination for continuous learning. Conversely, educational background plays a pivotal role in anxiety levels, with postgraduates expressing reduced concerns about AI-related errors due to stronger technical comprehension capabilities. Notably, frontline laboratory staff exhibit a profound worry about job displacement (32 %), higher than the 25 % reported by those in managerial roles. Multimodal AI models can mitigate the limitations of AI literacy among laboratory staff by integrating diverse data types (e.g., cell morphology images, cellular biomarkers, and biochemical parameters) into a unified analytical framework [20]. A multimodal system could combine convolutional neural networks (CNNs) to extract spatial features from blood smears, graph-based models to analyze cellular interaction networks, and regression algorithms to interpret biochemical trends [21], 22]. This integration reduces reliance on manual data synthesis, automating complex tasks such as identifying dysplastic cells in leukemia while correlating morphological anomalies with biochemical imbalances (e.g., elevated lactate dehydrogenase). To enhance accessibility, these models can be embedded into user-friendly platforms with intuitive interfaces – such as drag-and-drop image uploads and automated report generation – that abstract technical complexities. Additionally, incorporating explainable AI (XAI) components, like heatmaps highlighting critical cell features or natural language summaries of biochemical correlations, empowers laboratory personnel to validate outputs without requiring deep technical expertise. By streamlining workflows and providing contextualized insights, multimodal models bridge the gap between advanced AI capabilities and practical laboratory operations, fostering trust and adoption even among users with limited AI training.

Currently, AI knowledge reserves within laboratory teams are alarmingly inadequate, with only 10.8 % of teams demonstrating a robust command of AI, and a notably lower proportion of data scientists in tertiary hospitals compared to private medical institutions. This stark contrast is further highlighted by the 89.7 % of respondents who underscore an urgent demand for AI training. However, 47.2 % of laboratories face significant challenges in effectively implementing AI technologies, primarily due to inadequate IT support teams [23].

Discrepancies among stakeholders regarding the allocation of liability for AI-related medical errors are significant [24], [25], [26]. Survey findings reveal that 66.7 % of respondents advocate for shared responsibility between users and manufacturers, while some argue that manufacturers should bear sole accountability for products exhibiting design flaws. Clinicians lean towards assigning joint liability to manufacturers and healthcare institutions, asserting that manufacturers should assume primary responsibility if AI tools have undergone rigorous validation and comply with applicable standards [26], 27].

## Integration of HL7-FHIR/LIS interfaces and privacy-security considerations

The practical deployment of AI chatbots within laboratory settings requires robust integration with existing infrastructure, particularly through standardized interfaces such as Health Level 7 Fast Healthcare Interoperability Resources (HL7-FHIR) and Laboratory Information Systems (LIS) [28], 29]. HL7-FHIR enables interoperable data exchange between AI models and laboratory databases, ensuring real-time access to structured test results, patient histories, and diagnostic criteria [28], [29], [30], [31]. AI-driven interpretation of biochemical profiles could be enhanced by bidirectional communication with LIS, allowing automated flagging of critical values or contextual analysis of longitudinal data [31]. However, such integration demands robust cybersecurity protocols to safeguard sensitive health data. Basic privacy and security requirements include end-to-end encryption of data transmissions, role-based access controls compliant with regulations like General Data Protection Regulation (GDPR) and Health Insurance Portability and Accountability Act (HIPAA), and regular audits to detect vulnerabilities.

## The application of artificial intelligence chatbots in laboratory medicine

Currently, AI has achieved extensive application in areas such as radiology for image recognition, yet its utilization in laboratory medicine remains in its early stages. This is primarily due to the interpretation of laboratory reports involving a vast number of complex quantitative and qualitative variables, such as symptoms, medical history, and test results [32].These factors impose higher demands on the complexity and accuracy of AI models. Since the release of ChatGPT in 2022, it has garnered significant attention in the medical field. Relevant studies have investigated its performance in medical licensure exams, practicality in addressing patient inquiries, and capability in assisting doctors with clinical problem-solving, thereby showcasing the application potential of AI in laboratory medicine [33], [34], [35].

### Capabilities of ChatGPT in addressing clinical laboratory inquiries

Despite not undergoing specialized training on medical data, ChatGPT has demonstrated initial practicality in the medical field. A study by Munoz-Zuluaga et al. involved submitting 65 questions covering multiple topics to ChatGPT to assess its capabilities and accuracy in answering laboratory medicine-related questions [36]. The results showed that ChatGPT correctly answered 50.7 % of the questions, provided incomplete or partially correct responses for 23.1 %, delivered incorrect or misleading information for 16.9 %, and produced irrelevant answers for 9.3 %. Notably, correct answers were more concentrated in questions related to foundational medical or technical knowledge (59.1 %), while errors were more common in questions pertaining to laboratory procedures or regulations (31 %). Although GPT-4 shows significant improvements in accuracy, limitations in certain domains remain evident. A study by Girton et al. evaluated ChatGPT’s ability to address 49 real patient clinical laboratory questions on social media platforms such as Reddit and Quora, and compared these responses to those provided by medical students [37]. Reviewers tended to prefer ChatGPT’s answers over those from medical professionals. This highlights ChatGPT’s progress in handling complex clinical questions, while simultaneously revealing gaps in medical professional education in this area. Approximately half of the medical students’ answers were rated as “poor,” compared to less than 10 % of ChatGPT’s responses receiving similar ratings [37]. The limited emphasis on clinical laboratory knowledge in medical education may contribute to even experienced medical professionals struggling to provide high-quality answers to real-world questions.

Another study compared responses from three chatbots (ChatGPT, Gemini, and Le Chat) with those from certified physicians in online consultations [38]. Overall, chatbots outperformed online medical professionals in interpreting laboratory results. While online doctors ranked first in 60 % of cases, Gemini received lower scores in only 39 % of cases. ChatGPT demonstrated comparable quality and accuracy to human practitioners. However, chatbots showed higher tendencies for overestimating clinical severity (22–33 % incidence) compared to physicians’ 1.0 % overestimation rate. This highlights chatbots’ challenges in processing complex contextual information and interpreting laboratory data, with the lack of unified reference standards potentially leading to inconsistent interpretations of patient test data [38].

### Implementation of chatbots in medical laboratory diagnostics

Recent advancements in artificial intelligence have garnered significant research interest regarding biochemical data interpretation. Kaftan AN et al. demonstrated varying accuracy levels among AI models (Copilot, Gemini) in biochemical data analysis [39]. Copilot exhibited superior performance across all evaluation metrics, achieving statistically significant advantages in biochemical parameter interpretation compared to other models. This suggests Copilot’s enhanced data processing capabilities may stem from its sophisticated algorithmic architecture. While other models demonstrated suboptimal performance relative to Copilot, their domain-specific competencies retain practical relevance for targeted clinical applications [39].

The microbiology domain has emerged as a strategic focus for artificial intelligence (AI) integration due to its demanding requirements for diagnostic precision and rapid data processing. AI demonstrates transformative potential in enhancing clinical decision-making efficiency and optimizing infectious disease management, thereby improving patient outcomes. However, AI systems require further refinement in handling complex clinical scenarios and generating contextually appropriate responses to ensure clinical relevance [40]. Notably, ChatGPT-4.0 exhibits significant limitations when addressing Antimicrobial Susceptibility Testing (AST)-related inquiries [41], 42]. The model inappropriately recommended clindamycin for Enterococcus infections and failed to incorporate standard methodologies for polymyxin susceptibility assessment. Such erroneous guidance could misguide clinical practice, resulting in ineffective therapeutic regimens and potential patient harm.

Li Y et al. conducted a systematic evaluation of two LLMs, ChatGPT and Google Gemini, in addressing HBV-related clinical inquiries [43]. ChatGPT-4.0 demonstrated superior overall performance, achieving 80.8 % accuracy on evidence-based questions compared to Google Gemini (73.1 %). Domain-specific analysis revealed ChatGPT-4.0’s enhanced diagnostic utility in HBV serological interpretation, while Google Gemini provided more comprehensive descriptions of clinical manifestations. All models exhibited critical limitations in conveying HBV prevention strategies, particularly regarding vaccine information currency. Notably, only Google Gemini referenced the current consensus recommending neonatal hepatitis B vaccination within 12 h of birth. Furthermore, despite accuracy variations, both ChatGPT and Gemini responses exceeded the recommended 8th-grade readability level (Flesch-Kincaid grade 10.2–11.4), potentially compromising patient comprehension. These findings underscore the necessity for clinician verification of LLM outputs to ensure both interpretability and clinical validity. This comparative study provides crucial insights into model-specific strengths and limitations for evidence-based clinical implementation.

## Multidimensional challenges of healthcare chatbots in clinical practice

Although chatbots are designed and positioned as conversational tools rather than medical advisors or decision-support systems, they have demonstrated impressive capabilities in detecting and interpreting laboratory anomalies. This ability is particularly evident in their efficient processing and analysis of vast datasets, especially with advanced large language models like ChatGPT. However, these potential capabilities are accompanied by significant limitations, partly because these models have not been specifically trained or optimized for laboratory medicine reports.

### Operational challenges of ChatGPT in clinical laboratory interpretation

The European Federation of Clinical Chemistry and Laboratory Medicine (EFLM) Working Group evaluation reveals significant limitations in ChatGPT’s clinical utility despite its ability to generate “broadly correct and safety-relevant” laboratory result analyses [44]. Notably, ChatGPT fails to identify subclinical disease markers within normal reference ranges – for instance, it overlooks that elevated GGT may not conclusively indicate hepatic injury, and normal leukocyte subpopulation distributions don’t guarantee immune system integrity. This parameter-isolated analysis risks missing early pathology indicators. Critical deficiencies emerge in pre-analytical factor recognition. While ChatGPT appropriately flags diabetes risk with elevated glucose and HbA1c, it neglects crucial pre-collection variables (e.g., fasting status) when interpreting discordant results (elevated glucose with normal HbA1c). Moreover, ChatGPT demonstrates inadequate synthesis of multi-analyte profiles – interpretations of liver function markers (ALT, AST, bilirubin, GGT) lack essential clinical context integration required for comprehensive diagnostic reasoning [44]. Furthermore, ChatGPT fails to thoroughly analyze the health risks associated with test results. For example, in cases of severe anemia or lipid profile abnormalities, it merely advises patients to consult a doctor but does not clearly inform them of the potential clinical severity of these conditions, which could pose significant threats to patients’ health. Lastly, ChatGPT is unable to effectively distinguish between outliers and critical clinical threshold values. This limitation could potentially lead to medical errors in critical clinical scenarios. The inability to differentiate between abnormal values and emergency-level clinical values is particularly concerning, as such distinctions are crucial for timely and appropriate medical interventions.

### Diversity and variability of chatbots

In the current environment of diverse chatbot ecosystems, the variability among models and the challenges they pose in cross-study comparisons have become particularly significant. Generative AI models vary greatly in architecture, settings, and objectives, resulting in substantial differences in their performance and quality when generating content. Their architectural design and capabilities directly influence the accuracy and effectiveness of their outputs. Notably, there are significant differences in performance across different generative AI models for specific tasks. For instance, Bing has demonstrated the highest accuracy and specificity in predicting drug interactions, significantly outperforming Bard and ChatGPT-4, highlighting that certain models may have inherent advantages in specific application scenarios [45].

Furthermore, variability within the same model cannot be overlooked. The same model may produce inconsistent results under different configurations, input data, or generation strategies, especially in the public health domain, where such variability could have significant implications for decision-making [45], 46]. Differences in model performance not only affect the effectiveness of information dissemination but also directly influence user experience and satisfaction. For example, Bard has been found to provide the most easily understandable information regarding rhinoplasty, followed by ChatGPT and Bing [47]. Therefore, researchers and users should exercise careful selection when applying generative AI models to ensure their alignment with specific tasks and user needs.

### Risks associated with updates to chatbot versions

While continuous updates to chatbots aim to enhance their capabilities and performance, they also pose challenges in terms of data consistency, data quality, and reliability. Model updates may introduce new knowledge while risking reference to outdated data, particularly in rapidly evolving fields such as medicine. For example, the data used to train ChatGPT did not include the latest blood lead reference value of 3.5 μg/dL from the CDC for children. In contrast, CopyAI was able to provide accurate blood lead reference values, indicating that differences in training data updates across chatbots may lead to inconsistent judgment results [48]. Furthermore, updates do not always result in improved performance, as accuracy in certain tasks may decline. A study conducted by Stanford University and the University of California, Berkeley, found that while GPT-4.0 can provide more comprehensive information in certain areas, its accuracy in tasks such as mathematics, coding, and visual reasoning actually decreased [49]. This phenomenon underscores the importance of continuously evaluating the performance of LLMs, particularly in fields like laboratory medicine where accuracy is critical.

### Challenges of consistency in content generation by generative AI models

The consistency of chatbot output in content generation deserves close attention, particularly in specialized fields such as medicine. Research indicates that minor differences in the wording of prompts or background information can lead to significant variations in generated content, potentially affecting reliability in practical applications [11], 50], 51]. A study by Kochanek and colleagues demonstrated that the consistency of responses from GPT-4.0 when answering the same question over four days was 85–88 %, respectively, highlighting the influence of uncertainties in content generation [52]. In the medical field, where high consistency in output is critical, variability in answers could pose notable risks. Further evaluation of the reproducibility and accuracy of ChatGPT in laboratory medicine is essential to ensure its reliability as a reference in medical applications [37].

Moreover, generative AI models can be influenced by cultural and socio-cultural biases inherent in their training data, leading to inconsistencies in generated content across different cultural or linguistic contexts. For instance, Wang and colleagues found that ChatGPT performed significantly better in English than in Chinese during the Taiwanese Pharmacy Licensing Examination [53]. Alfertshofer and colleagues further observed that ChatGPT exhibited substantial differences in performance across six countries in medical licensing examinations, highlighting the impact of national and linguistic factors [54].These findings underscore the need for deeper investigation and optimization of generative AI models in multilingual and cross-cultural settings to enhance their adaptability and accuracy in diverse scenarios.

### Challenges in the quality of health information dissemination

Chatbots are playing an increasingly important role in the dissemination of health information, but the clarity, conciseness, and relevance of their responses require careful attention [37]. Clarity demands that the generated content is easy to understand, avoiding the use of complex medical jargon so that the general public can grasp the key points effortlessly. Simultaneously, conciseness requires straightforward communication without unnecessary length, enhancing the appropriateness of health information and aiding the public in improving their health literacy, thereby enabling them to make informed health decisions. Additionally, the relevance of AI-generated content is crucial. Accurate and relevant information helps prevent misunderstandings, particularly in scenarios where patients cannot access face-to-face explanations from doctors, avoiding unnecessary anxiety or health risks caused by misinformation [55]. Prioritizing the relevance of information prevents information overload, ensuring the clear delivery of critical health messages. Information unrelated to health inquiries may disrupt people’s understanding of their health conditions, increasing their confusion when dealing with personal health issues.

However, the dissemination of health information via AI must balance the differing needs of patients and medical professionals. Patients typically prefer short, direct answers to quickly comprehend key information, while medical professionals tend to favor detailed and comprehensive responses. This divergence of needs creates a double-edged sword: while abundant information aids professionals in thorough analysis, it may overwhelm ordinary users, leading to comprehension difficulties and anxiety. Regardless of the presentation method, ensuring the completeness of information is paramount. The absence of necessary details may lead to misdiagnosis or inaccurate self-diagnosis, exacerbating health risks. For instance, patients with limited health literacy may struggle to interpret laboratory test results accurately, such as identifying high cholesterol levels in lipid panels, potentially leading to severe health issues like heart disease or stroke [44]. Therefore, enhancing patients’ ability to understand laboratory test results is of utmost importance.

### Risks of chatbots providing “hallucination” information

The application of chatbots in the dissemination of medical information is becoming increasingly widespread, but it has also raised significant concerns regarding the risk of misinformation. While generative AI tools like GPT offer certain advantages in providing information, their inability to explain decision-making processes hinders their ability to effectively identify and correct biases or errors within the model. In the medical field, AI-generated misinformation can lead to severe consequences, including incorrect self-diagnoses, delayed medical care, the spread of potentially harmful diseases, and a loss of public trust in healthcare professionals and institutions. For instance, GPT has provided inaccurate descriptions of FDA-approved high-sensitivity troponin point-of-care testing devices. Such “hallucination” responses are not isolated incidents, highlighting the limitations of generative AI models in ensuring the accuracy of information [56], 57]. Therefore, ensuring the accuracy, reliability, and trustworthiness of AI-generated medical information is of paramount importance. Developers must prioritize this objective during the design and training of AI models, as existing studies demonstrate that the generation of inaccurate information is a widespread phenomenon in such tools.

### Legal and ethical issues in chatbot training data

Legal and ethical issues surrounding chatbot training data are critical concerns in the development of generative artificial intelligence (AI) models [58], [59], [60], [61]. These issues not only pertain to the origin of the data but also involve its usage and acceptability, directly impacting the transparency and credibility of the model. Data transparency is of particular importance, ensuring that users and researchers can understand the methods of data collection and utilization, thereby validating the reliability and scientific integrity of the model. Additionally, ethical concerns must not be overlooked, especially when using copyrighted materials and clinical data, as it is imperative to ensure legal and compliant data acquisition and usage while safeguarding the privacy rights of data providers.

Generative AI models like ChatGPT are trained on internet-based data, inevitably inheriting biases present in the training data and reflecting them in their outputs [62]. Studies have shown that LLMs exhibit biases related to gender, race, and other social factors, which not only compromise the fairness of model outputs but also exacerbate social inequalities. These biases are closely linked to issues such as data gaps, insufficient sample sizes, and inherent biases in foundational datasets [63]. If the training data is older, the potential biases and errors may be amplified in subsequent model outputs [64]. This phenomenon is particularly evident in existing records, especially in key areas such as randomized design, further highlighting the potential biases in query selection processes.

## EU AI act and FDA clinical-decision-support (CDS) guidance

The European Union’s Artificial Intelligence Act (AI Act), effective from July 12, 2024, establishes the world’s first comprehensive regulatory framework for AI, prioritizing ethical development, fundamental rights protection, and human-centric innovation [65]. Adopting a risk-based approach, the Act categorizes AI systems into four tiers: unacceptable risk (banned), high-risk (e.g., medical diagnostics, subject to strict safety, transparency, and accountability requirements), general-purpose AI with systemic risk (transparency obligations), and low/no-risk (minimal regulation) [66]. In healthcare, stringent standards for high-risk medical AI emphasize rigorous testing, bias mitigation, and post-market monitoring, though gaps persist in addressing patient rights and accountability for low-risk applications. Challenges include uneven implementation across member states, ambiguous liability frameworks, and evolving limitations in detecting AI-generated content [67]. The Act also addresses generative AI’s copyright concerns, mandating transparency in training data and safeguarding intellectual property, while exempting pre-market research [66]. Extraterritorial impacts akin to the GDPR is expected, reshaping global AI practices and necessitating AI ethics education. Balancing regulation with innovation, the AI Act aims to foster trustworthy AI but requires clearer guidelines, stakeholder collaboration, and tailored healthcare standards to ensure equitable access and patient safety.

Currently, no LLM is authorized by the FDA as a CDS device. Research has evaluated whether LLMs can adhere to regulations in the context of clinical emergencies. The findings revealed that while LLMs provided appropriate preventive care recommendations, 100 % of responses from GPT-4 and 52 % from Llama-3 were noncompliant in time-critical scenarios, yielding device-like support by suggesting specific diagnoses and treatments [68]. Despite prompts based on FDA guidance, compliance was not consistently achieved. These results highlight the urgent need for new regulatory frameworks to address the unique challenges posed by generative AI in healthcare, as current guidelines are insufficient to prevent unauthorized device-like outputs from LLMs.

## Establishing a comprehensive evaluation framework to address the challenges of AI chatbots in healthcare

In response to the risks posed by AI chatbots in the medical field, it is imperative to develop a comprehensive evaluation framework. The first step in ensuring the quality of AI outputs involves establishing strict evaluation criteria that encompass the accuracy, reliability, and relevance of information. Additionally, regular performance testing helps identify system flaws and ensures that AI models remain aligned with updates in medical knowledge. Feedback mechanisms, which gather input from users and professionals, provide valuable data for continuous improvement. The inclusion of professional review mechanisms is equally crucial. The involvement of clinical experts further enhances the reliability and scientific integrity of AI systems, ensuring that the generated information aligns with medical ethics and practice standards.

To achieve this goal, various tools and guidelines have been developed to assist in evaluating and improving the quality of health information [69], [70], [71]. However, existing health information evaluation tools are not specifically tailored to assess the quality of health-related content generated by AI models. Ensuring the reliability and accuracy of AI in medical laboratory environments necessitates the use of standardized evaluation tools. Currently, the “METRICS” and “CLEAR” tools are widely recognized as effective means of assessing AI-generated health information [72], 73].

The METRICS checklist provides a framework for designing and reporting standardized AI research, covering nine key themes: Model, Evaluation, Timing, Range/Randomization, Individual Factors, Count, and Specificity of Prompts and Language. These themes aim to comprehensively analyze the design and operational status of AI models, ensuring the scientific rigor and robustness of their output. In contrast, the CLEAR tool focuses on systematically evaluating multiple dimensions of AI-generated content, including five key criteria: Completeness of Content, Lack of False Information in Content, Evidence Supporting the Content, Appropriateness of the Content, and Relevance. The specific meanings of each criterion are detailed in Table 1 and Table 2, providing evaluators with a clear guide to ensure that AI-generated content meets predefined quality standards.

**Table 1:** Entries for METRICS.

Tool type	Item content	Limitations	Scale rating
METRICS includes nine items:			The items of METRICS is scored using a 5-point likert scale as follows: 5=excellent, 4=very good, 3=good, 2=satisfactory, and 1=suboptimal.
(1) Model	(1) Model: The generative AI-based tool(s) used in the included record should explicitly mention the exact settings for each tool.	Limited literature scope: The literature was retrieved using only a single keyword, which may result in omissions.	
(2) Evaluation	(2) Evaluation: The approach to assessing content quality balances objectivity (unbiased findings) with subjectivity.	(2) Database and language bias: The English literature from databases such as scopus, PubMed, and google scholar may introduce potential selection bias.	
(3) Timing	(3) Timing: The exact timing and duration of the generative AI model testing.	(3) Insufficient topics: Due to the limitations of the author team (single-disciplinary background), relevant cross-disciplinary topics may have been overlooked.	
(4) Transparency	(4) Transparency: Transparency of data sources (including permissions for copyrighted content).	(4) Subjectivity risk: The selection of topics and the evaluation of authors (METRICS scoring) may involve subjective judgments.	
(5) Range	(5) Range: The scope of tested topics (single/multiple related/unrelated topics; breadth of intra- and inter-topic queries).	(5) Equal weighting in scoring: All METRICS adopted the same weight, disregarding the differences in importance among various indicators. Coverage limitations of models: The focus was solely on ChatGPT, bing, and bard, without considering other generative models (such as claude).	
(6) Randomization	(6) Randomization: Degree of randomization in topic selection to mitigate bias.		
(7) Individual factors	(7) Individual: Subjective role in content evaluation and interrater reliability (agreement/disagreement).		
(8) Count	(8) Count: Number of queries executed per model (sample size).		
(9) Specificity of prompts and language	(9) Specificity: Prompts: Exact phrasing, feedback mechanisms, and learning loops. Language: The language(s) used in testing, including cultural considerations (e.g., linguistic and cultural appropriateness).		

**Table 2:** Entries for CLEAR.

Tool type	Item content	Limitations	Scale rating
CLEAR includes five items:			The items of CLEAR is scored using a 5-point likert scale as follows: 5=excellent, 4=very good, 3=good, 2=satisfactory, and 1=suboptimal.
(1) Completeness of content	(1) Is the content sufficient? Completeness means that the information is generated in an optimal manner, not too excessive and not deficient.	(1) Sample limitations: The assessment of the CLEAR tool relies solely on a small number of homogeneous healthcare professionals, which introduces bias; the test data consists of artificially generated statements, lacking contextual validation.	
(2) Lack of false information in the content	(2) Is the content accurate? The generation of incorrect health information by AI tools can lead to serious negative consequences.	(2) Insufficient tool validation: The CLEAR tool has not been compared with other information assessment tools, such as DISC, to clarify its strengths and weaknesses; the effectiveness of the tool should be further confirmed through testing on broader, especially contentious health topics.	
(3) Evidence supporting the content	(3) Is the content evidence-based? This means that the health information provided by the AI model should be based on the latest scientific advancements and avoid bias, misinformation, or false information.	(3) Impact of AI model dynamics: AI models are continually updated and iterated (e.g., changes from GPT-4 to GPT-5 may yield different results with the same input over time); model performance is sensitive to the design of prompts, and variations in prompts can lead to result discrepancies.	
(4) Appropriateness of the content	(4) Is the content clear, concise, and easy to understand? The content should also provide a single, clear explanation and be well-organized in a logical sequence to facilitate understanding.	(4) Limited external applicability: The current conclusions are based on assessments from a limited disciplinary background, indicating a need for validation involving multi-disciplinary experts (such as AI developers and patient representatives).	
(5) Relevance	(5) Is the content free from irrelevant information? Relevance refers to the necessity of prviding accurate and pertinent health content.		

In a study by Malik Sallam et al., three generative AI models (ChatGPT, Microsoft Bing, and Google Bard) were evaluated using the METRICS tool [72]. The overall mean METRICS score across these models was 3.0 (SD 0.58). Analyzing individual criteria, the “Model” criterion received the highest average score, followed by “Specificity”. Conversely, the lowest scores were observed for the “Randomization” criterion (classified as suboptimal) and the “Individual factors” criterion (classified as satisfactory). Separately, the five AI models were assessed using the CLEAR tool across five distinct topics [73]. Microsoft Bing achieved the highest average CLEAR score (mean: 24.4 ± 0.42), followed by ChatGPT-4 (mean: 23.6 ± 0.96), Google Bard (mean: 21.2 ± 1.79), and finally ChatGPT-3.5 (mean: 20.6 ± 5.20).

While the METRICS and CLEAR tools have contributed to the evaluation of AI-generated content, they also have certain limitations (as detailed in Table 1 and Table 2). For example, these tools may fail to cover all variables in specific situations or exhibit limited applicability in certain specialized domains. Therefore, these limitations require validation through further research to confirm their effectiveness and suitability in practical applications. By gaining a deeper understanding of and addressing these limitations, we can enhance the credibility of AI-generated health information, providing a solid foundation for medical decision-making.

## Conclusions and outlook

**Figure 1:**
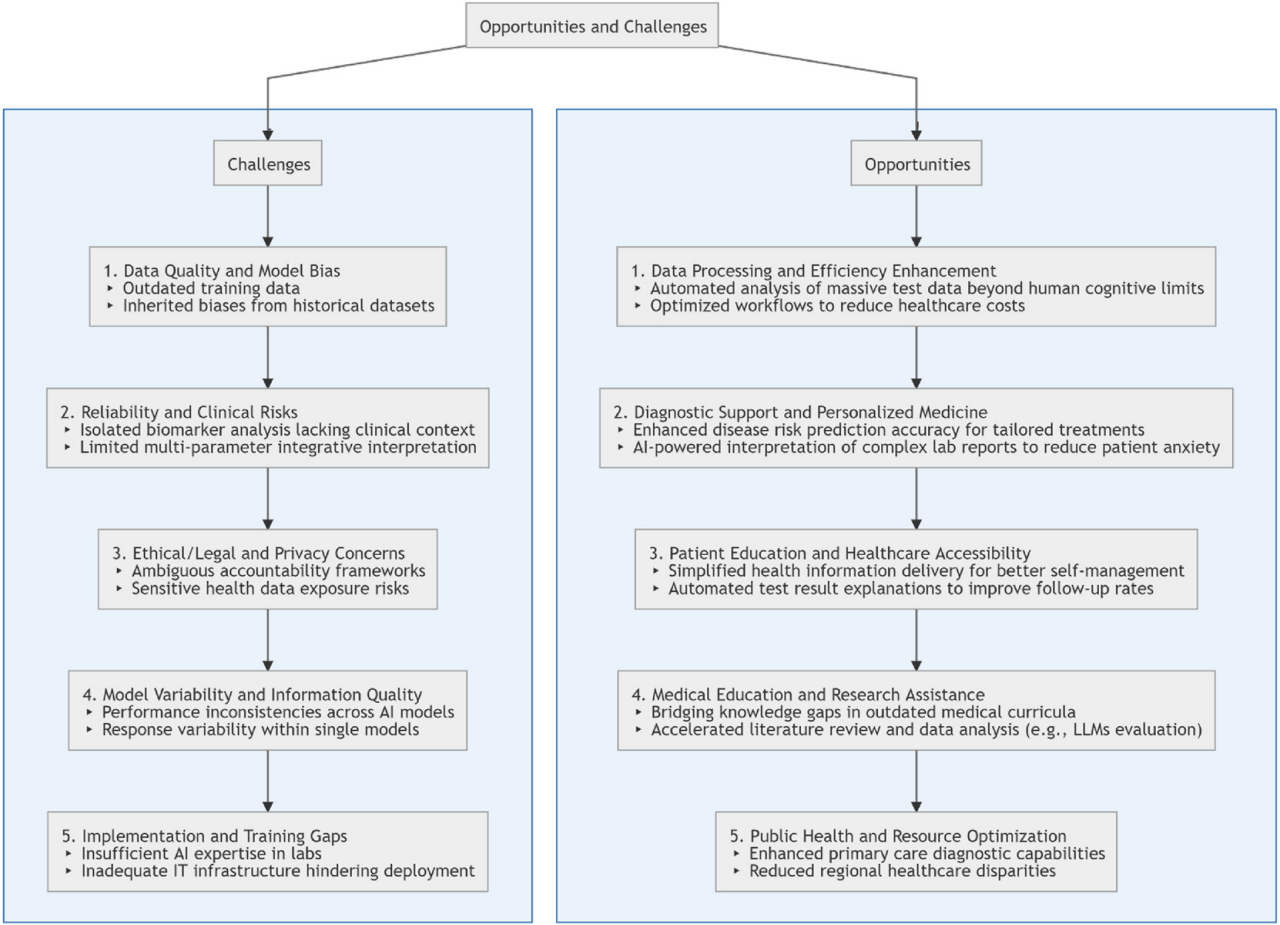
Diagram of opportunities and challenges.

Although AI chatbots face limitations such as the need for advanced knowledge, insufficient reasoning abilities, and limited capacity for in-depth analysis of patient information, their potential in disease diagnosis and laboratory medicine should not be overlooked, as illustrated in Figure 1, which provides a concise summary of the opportunities and challenges associated with chatbots. In the field of laboratory medicine, AI chatbots like GPT are not only capable of providing accurate information but may also reach or exceed the response level of medical professionals in certain key areas [37]. Medical education is a time-intensive process with slow updates, making it challenging for students to grasp all critical points. Therefore, AI chatbots have the potential to serve as important auxiliary tools in medical education, helping to bridge the gap in students’ knowledge acquisition. However, creative problem-solving remains a unique strength of human beings. To fully leverage AI’s potential in medicine, collaboration between AI developers and healthcare professionals is crucial.

Laboratory medicine is a rapidly evolving field, with new technologies and testing methods continuously being developed and integrated into clinical practice. If the limitations of AI chatbots can be effectively addressed, they could become indispensable tools for clinicians and laboratory technicians in their daily work. Overcoming existing challenges could lead to improved diagnostic precision, enhanced patient care quality, and the promotion of personalized medicine. Collaboration between AI and human medical experts will be the foundation for the future development of laboratory medicine.

## References

[1] Kaul V, Enslin S, Gross SA (2020). History of artificial intelligence in medicine. Gastrointest Endosc.

[2] Ferraro S, Panteghini M (2017). The role of laboratory in ensuring appropriate test requests. Clin Biochem.

[3] Lavoie-Gagne O, Woo JJ, Williams RJ, Nwachukwu BU, Kunze KN, Ramkumar PN (2025). Artificial intelligence as a tool to mitigate administrative burden, optimize billing, reduce insurance and credentialing-related expenses, and improve quality assurance within healthcare systems. Arthroscopy.

[4] Johri S, Jeong J, Tran BA, Schlessinger DI, Wongvibulsin S, Barnes LA (2025). An evaluation framework for clinical use of large language models in patient interaction tasks. Nat Med.

[5] Giardina TD, Baldwin J, Nystrom DT, Sittig DF, Singh H (2018). Patient perceptions of receiving test results via online portals: a mixed-methods study. J Am Med Inform Assoc.

[6] Bar-Lev S, Beimel D (2020). Numbers, graphs and words - do we really understand the lab test results accessible via the patient portals?. Isr J Health Pol Res.

[7] Chu SKW, Huang H, Wong WNM, Ginneken WFV, Hung MY (2018). Quality and clarity of health information on Q&A sites. Libr Inf Sci Res.

[8] He Z, Bhasuran B, Jin Q, Tian S, Hanna K, Shavor C (2024). Quality of answers of generative large language models versus peer users for interpreting laboratory test results for lay patients: evaluation study. J Med Internet Res.

[9] (2023). Will ChatGPT transform healthcare?. Nat Med.

[10] Eysenbach G (2023). The role of ChatGPT, generative language models, and artificial intelligence in medical education: a conversation with ChatGPT and a call for papers. JMIR Med Educ.

[11] Meskó B (2023). Prompt engineering as an important emerging skill for medical professionals: tutorial. J Med Internet Res.

[12] Hristidis V, Ruggiano N, Brown EL, Ganta SRR, Stewart S (2023). ChatGPT vs google for queries related to dementia and other cognitive decline: comparison of results. J Med Internet Res.

[13] Sallam M (2023). ChatGPT utility in healthcare education, research, and practice: systematic review on the promising perspectives and valid concerns. Healthcare.

[14] Rajpurkar P, Chen E, Banerjee O, Topol EJ (2022). AI in health and medicine. Nat Med.

[15] Shahsavar Y, Choudhury A (2023). User intentions to use ChatGPT for self-diagnosis and health-related purposes: cross-sectional survey study. JMIR Hum Factors.

[16] Kopanitsa G (2022). Study of patients’ attitude to automatic interpretation of laboratory test results and its influence on follow-up rate. BMC Med Inform Decis Mak.

[17] Sarwar S, Dent A, Faust K, Richer M, Djuric U, Van Ommeren R (2019). Physician perspectives on integration of artificial intelligence into diagnostic pathology. NPJ Digit Med.

[18] Ardon O, Schmidt RL (2020). Clinical laboratory employees’ attitudes toward artificial intelligence. Lab Med.

[19] Oh S, Kim JH, Choi SW, Lee HJ, Hong J, Kwon SH (2019). Physician confidence in artificial intelligence: an online Mobile survey. J Med Internet Res.

[20] Tarmissi K, Alsamri J, Maashi M, Asiri MM, Yahya AE, Alkharashi A (2025). Multimodal representations of transfer learning with snake optimization algorithm on bone marrow cell classification using biomedical histopathological images. Sci Rep.

[21] Matek C, Krappe S, Münzenmayer C, Haferlach T, Marr C (2021). Highly accurate differentiation of bone marrow cell morphologies using deep neural networks on a large image data set. Blood.

[22] Durant TJS, Olson EM, Schulz WL, Torres R (2017). Very deep convolutional neural networks for morphologic classification of erythrocytes. Clin Chem.

[23] Cadamuro J, Carobene A, Cabitza F, Debeljak Z, De Bruyne S, van Doorn W (2025). A comprehensive survey of artificial intelligence adoption in European laboratory medicine: current utilization and prospects. Clin Chem Lab Med.

[24] Yu S, Jeon BR, Liu C, Kim D, Park HI, Park HD (2024). Laboratory preparation for digital medicine in healthcare 4.0: an investigation into the awareness and applications of big data and artificial intelligence. Ann Lab Med.

[25] Khullar D, Casalino LP, Qian Y, Lu Y, Chang E, Aneja S (2021). Public vs physician views of liability for artificial intelligence in health care. J Am Med Inform Assoc.

[26] Abràmoff MD, Tobey D, Char DS (2020). Lessons learned about autonomous AI: finding a safe, efficacious, and ethical path through the development process. Am J Ophthalmol.

[27] Bazoukis G, Hall J, Loscalzo J, Antman EM, Fuster V, Armoundas AA (2022). The inclusion of augmented intelligence in medicine: a framework for successful implementation. Cell Rep Med.

[28] Wen Y, Choo VY, Eil JH, Thun S, Pinto Dos Santos D, Kast J (2025). Exchange of quantitative computed tomography assessed body composition data using fast healthcare interoperability resources as a necessary step toward interoperable integration of opportunistic screening into clinical practice: methodological development study. J Med Internet Res.

[29] Dolin RH, Heale BSE, Alterovitz G, Gupta R, Aronson J, Boxwala A (2023). Introducing HL7 FHIR genomics operations: a developer-friendly approach to genomics-EHR integration. J Am Med Inform Assoc.

[30] Vorisek CN, Lehne M, Klopfenstein SAI, Mayer PJ, Bartschke A, Haese T (2022). Fast healthcare interoperability resources (FHIR) for interoperability in health research: systematic review. JMIR Med Inform.

[31] Tran DM, Thanh Dung N, Minh Duc C, Ngoc Hon H, Minh Khoi L, Phuc Hau N (2025). Status of digital health technology adoption in 5 Vietnamese hospitals: cross-sectional assessment. JMIR Form Res.

[32] Burnside ES, Grist TM, Lasarev MR, Garrett JW, Morris EA (2025). Artificial intelligence in radiology: a leadership survey. J Am Coll Radiol.

[33] Takagi S, Watari T, Erabi A, Sakaguchi K (2023). Performance of GPT-3.5 and GPT-4 on the Japanese medical licensing examination: comparison study. JMIR Med Educ.

[34] Wang H, Wu W, Dou Z, He L, Yang L (2023). Performance and exploration of ChatGPT in medical examination, records and education in Chinese: pave the way for medical AI. Int J Med Inf.

[35] Lim ZW, Pushpanathan K, Yew SME, Lai Y, Sun CH, Lam JSH (2023). Benchmarking large language models’ performances for myopia care: a comparative analysis of ChatGPT-3.5, ChatGPT-4.0, and google bard. EBioMedicine.

[36] Munoz-Zuluaga C, Zhao Z, Wang F, Greenblatt MB, Yang HS (2023). Assessing the accuracy and clinical utility of ChatGPT in laboratory medicine. Clin Chem.

[37] Girton MR, Greene DN, Messerlian G, Keren DF, Yu M (2024). ChatGPT vs medical professional: analyzing responses to laboratory medicine questions on social media. Clin Chem.

[38] Meyer A, Soleman A, Riese J, Streichert T (2024). Comparison of ChatGPT, Gemini, and Le Chat with physician interpretations of medical laboratory questions from an online health forum. Clin Chem Lab Med.

[39] Kaftan AN, Hussain MK, Naser FH (2024). Response accuracy of ChatGPT 3.5 copilot and gemini in interpreting biochemical laboratory data a pilot study. Sci Rep.

[40] Sallam M, Al-Salahat K, Al-Ajlouni E (2023). ChatGPT performance in diagnostic clinical microbiology laboratory-oriented case scenarios. Cureus.

[41] Carey RB, Bhattacharyya S, Kehl SC, Matukas LM, Pentella MA, Salfinger M (2018). Practical guidance for clinical microbiology laboratories: implementing a quality management system in the medical microbiology laboratory. Clin Microbiol Rev.

[42] Genzen JR, Tormey CA (2011). Pathology consultation on reporting of critical values. Am J Clin Pathol.

[43] Li Y, Huang CK, Hu Y, Zhou XD, He C, Zhong JW (2025). Exploring the performance of large language models on hepatitis B infection-related questions: a comparative study. World J Gastroenterol.

[44] Cadamuro J, Cabitza F, Debeljak Z, De Bruyne S, Frans G, Perez SM (2023). Potentials and pitfalls of ChatGPT and natural-language artificial intelligence models for the understanding of laboratory medicine test results. An assessment by the european Federation of clinical chemistry and laboratory medicine (EFLM) working group on Artificial intelligence (WG-AI). Clin Chem Lab Med.

[45] Al-Ashwal FY, Zawiah M, Gharaibeh L, Abu-Farha R, Bitar AN (2023). Evaluating the sensitivity, specificity, and accuracy of ChatGPT-3.5, ChatGPT-4, bing AI, and bard against conventional drug-drug interactions clinical tools. Drug Healthc Patient Saf.

[46] Baglivo F, De Angelis L, Casigliani V, Arzilli G, Privitera GP, Rizzo C (2023). Exploring the possible use of AI chatbots in public health education: feasibility study. JMIR Med Educ.

[47] Seth I, Lim B, Xie Y, Cevik J, Rozen WM, Ross RJ (2023). Comparing the efficacy of large language models ChatGPT, BARD, and bing AI in providing information on rhinoplasty: an observational study. Aesthet Surg J Open Forum.

[48] Abusoglu S, Serdar M, Unlu A, Abusoglu G (2024). Comparison of three chatbots as an assistant for problem-solving in clinical laboratory. Clin Chem Lab Med.

[49] Lingjiao Chen MZ, James Z (2023). <How is chatgpt’s behavior changing over time_.pdf>. Harv Data Sci Rev.

[50] Giray L (2023). Prompt engineering with ChatGPT: a guide for academic writers. Ann Biomed Eng.

[51] Khlaif ZN, Mousa A, Hattab MK, Itmazi J, Hassan AA, Sanmugam M (2023). The potential and concerns of using AI in scientific research: ChatGPT performance evaluation. JMIR Med Educ.

[52] Kochanek K, Skarzynski H, Jedrzejczak WW (2024). Accuracy and repeatability of ChatGPT based on a set of multiple-choice questions on objective tests of hearing. Cureus.

[53] Wang YM, Shen HW, Chen TJ (2023). Performance of ChatGPT on the pharmacist licensing examination in Taiwan. J Chin Med Assoc.

[54] Alfertshofer M, Hoch CC, Funk PF, Hollmann K, Wollenberg B, Knoedler S (2024). Sailing the seven seas: a multinational comparison of chatgpt’s performance on medical licensing examinations. Ann Biomed Eng.

[55] Zikmund-Fisher BJ, Scherer AM, Witteman HO, Solomon JB, Exe NL, Fagerlin A (2018). Effect of harm anchors in visual displays of test results on patient perceptions of urgency about near-normal values: experimental study. J Med Internet Res.

[56] van Dis EAM, Bollen J, Zuidema W, van Rooij R, Bockting CL (2023). ChatGPT: five priorities for research. Nature.

[57] Sanderson K (2023). GPT-4 is here: what scientists think. Nature.

[58] Murdoch B (2021). Privacy and artificial intelligence: challenges for protecting health information in a new era. BMC Med Ethics.

[59] Mijwil MM, Aljanabi M, Ali AH (2023). ChatGPT: exploring the role of cybersecurity in the protection of medical information. Mesopotamian J CyberSecurity.

[60] Chen Z (2023). Ethics and discrimination in artificial intelligence-enabled recruitment practices. Palgrave Commun.

[61] Wang C, Liu S, Yang H, Guo J, Wu Y, Liu J (2023). Ethical considerations of using ChatGPT in health care. J Med Internet Res.

[62] Liang P, Wu C, Morency LP, Salakhutdinov R (2021). Towards understanding and mitigating social biases in language models. PMLR.

[63] Gianfrancesco MA, Tamang S, Yazdany J, Schmajuk G (2018). Potential biases in machine learning algorithms using electronic health record data. JAMA Intern Med.

[64] Tianyu Gao HY, Jiatong Y, Danqi C (2023). <Enabling large language models to generate text with citations.pdf>. ArXiv.

[65] Gasser U (2023). An EU landmark for AI governance. Science.

[66] März M, Himmelbauer M, Boldt K, Oksche A (2024). Legal aspects of generative artificial intelligence and large language models in examinations and theses. GMS J Med Educ.

[67] Alvarado A (2025). Lessons from the EU AI act. Patterns (N Y).

[68] Weissman G, Mankowitz T, Kanter G (2024). Large language model non-compliance with FDA guidance for clinical decision support devices. Res Sq.

[69] Charnock D, Shepperd S, Needham G, Gann R (1999). DISCERN: an instrument for judging the quality of written consumer health information on treatment choices. J Epidemiol Community Health.

[70] Baur C, Prue C (2014). The CDC clear communication index is a new evidence-based tool to prepare and review health information. Health Promot Pract.

[71] DeWalt DA, Broucksou KA, Hawk V, Brach C, Hink A, Rudd R (2011). Developing and testing the health literacy universal precautions toolkit. Nurs Outlook.

[72] Sallam M, Barakat M, Sallam M (2024). A preliminary checklist (METRICS) to standardize the design and reporting of studies on generative artificial intelligence-based models in health care education and practice: Development study involving a literature review. Interact J Med Res.

[73] Sallam M, Barakat M, Sallam M (2023). Pilot testing of a tool to standardize the assessment of the quality of health information generated by artificial intelligence-based models. Cureus.

